# Revisiting the Pathoetiology of Multiple Sclerosis: Has the Tail Been Wagging the Mouse?

**DOI:** 10.3389/fimmu.2020.572186

**Published:** 2020-09-29

**Authors:** Monokesh K. Sen, Mohammed S. M. Almuslehi, Peter J. Shortland, Jens R. Coorssen, David A. Mahns

**Affiliations:** ^1^School of Medicine, Western Sydney University, Penrith, NSW, Australia; ^2^Department of Physiology, College of Veterinary Medicine, University of Diyala, Baqubah, Iraq; ^3^School of Science, Western Sydney University, Penrith, NSW, Australia; ^4^Departments of Health Sciences and Biological Sciences, Faculties of Applied Health Sciences and Mathematics & Science, Brock University, St. Catharines, ON, Canada

**Keywords:** inside-out, outside-in, immune response, oligodendrocytosis, cuprizone, experimental autoimmune encephalomyelitis, demyelination, CNS disorder

## Abstract

Multiple Sclerosis (MS) is traditionally considered an autoimmune-mediated demyelinating disease, the pathoetiology of which is unknown. However, the key question remains whether autoimmunity is the initiator of the disease (outside-in) or the consequence of a slow and as yet uncharacterized cytodegeneration (oligodendrocytosis), which leads to a subsequent immune response (inside-out). Experimental autoimmune encephalomyelitis has been used to model the later stages of MS during which the autoimmune involvement predominates. In contrast, the cuprizone (CPZ) model is used to model early stages of the disease during which oligodendrocytosis and demyelination predominate and are hypothesized to precede subsequent immune involvement in MS. Recent studies combining a boost, or protection, to the immune system with disruption of the blood brain barrier have shown CPZ-induced oligodendrocytosis with a subsequent immune response. In this Perspective, we review these recent advances and discuss the likelihood of an inside-out vs. an outside-in pathoetiology of MS.

## Introduction

Sir Robert Carswell, in his account of spinal cord lesions in humans, was the first to described demyelination in the human central nervous system (CNS) in 1838. These lesions were accompanied by atrophy and discolouration that were termed “*a peculiar disease*,” (1) but made no attribution to Multiple Sclerosis (MS). Twenty-eight years later, in 1866, the French neurologist Jean-Martin Charcot first described these lesions as MS, “*la Sclerose en plaques disseminee*,” and delineated it from other neurological diseases such as neurosyphilis, epilepsy, and progressive amyotrophies. Charcot also established diagnostic criteria based on the loss of myelin, thickening of small blood vessels, and the presence of fatty macrophages around vessels. In addition, he described that axons were more resistant to injury than myelin and the disability was linked to the axonal damage [reviewed by (2)]. Over 150 years (1866–2020) from the initial description, demyelination in the CNS remains the central McDonald criterion for MS in current diagnostic schemes (i.e., two or more separate episodes of hyperintense demyelinating lesions in at least two or more separate CNS locations) (3). Although the presence of oligoclonal bands in the cerebrospinal fluid of MS patients is used as an additional diagnostic criterion (3), the antigen(s) against which these antibodies react remain poorly defined and are *not* disease-specific (4). For example, oligoclonal bands can be found in patients with other diseases such as neurosyphilis (5), subacute sclerosing panencephalitis (6), and falciparum malaria (7). Furthermore, it is not unusual for patients to be diagnosed with MS in the absence of oligoclonal bands (8, 9). These observations suggest that demyelination is the primary criterion (disseminated in time and space) to diagnose MS patients and the involvement of immune cells in MS is not necessary for the diagnosis.

**Figure 1:**
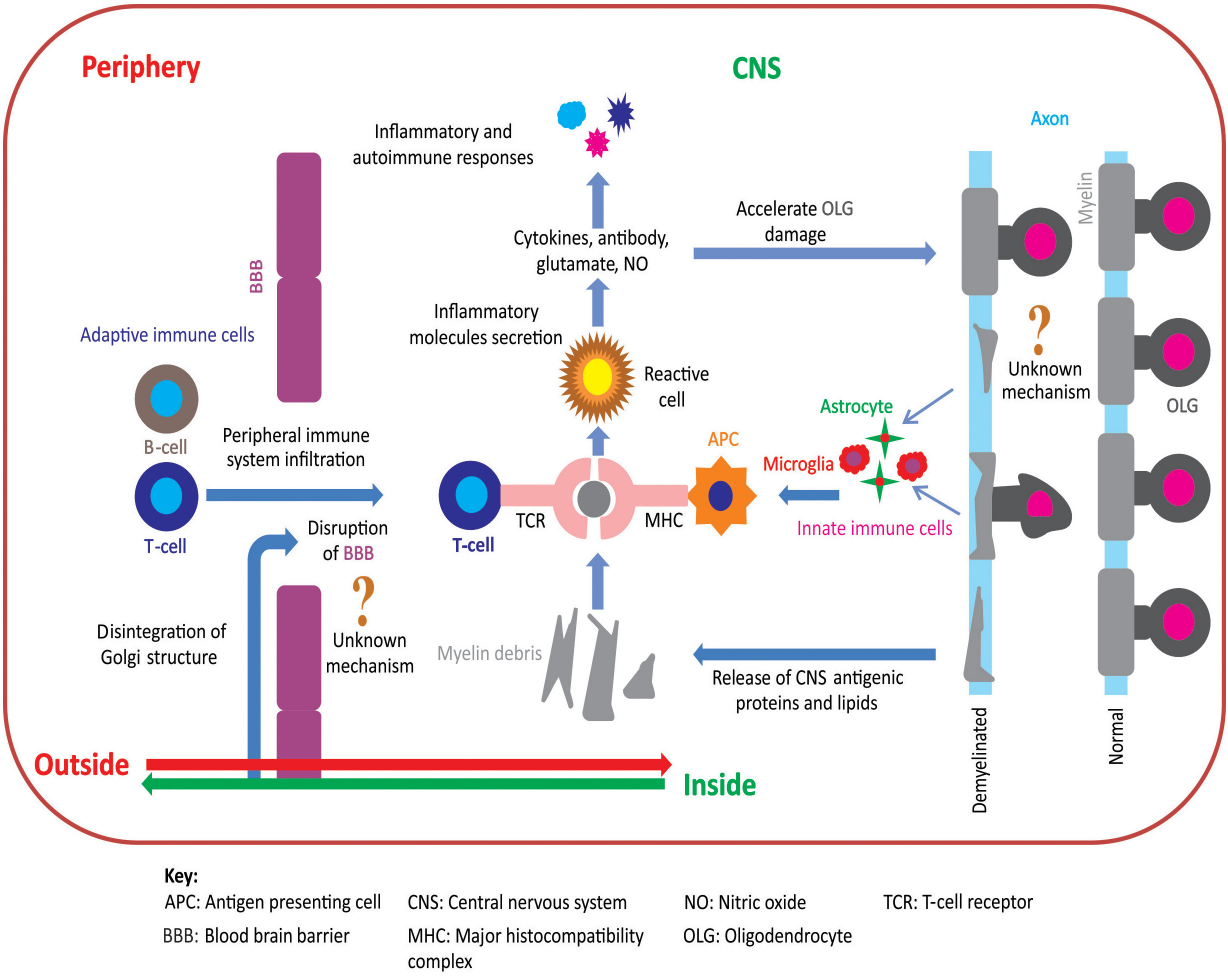
Pathoetiology of oligodendrocytosis and autoimmunity in MS. There are two principal competing hypotheses of MS pathoetiology. In the “outside-in” hypothesis, an unknown trigger activates peripheral T- and/or B-cells and leads to an infiltration of these cells into the CNS via an apparently dysfunctional blood brain barrier (BBB). T-cells attack myelin in the CNS causing oligodendrocytes to degenerate, resulting in myelin loss and the release of myelin debris. Innate immune cells (e.g., microglia) engulf the myelin debris and act as antigen presenting cells to T-cells that then exacerbate the process of oligodendrocyte damage and demyelination. In contrast, in the “inside-out” hypothesis, oligodendrocyte degeneration is initially triggered by internal metabolic dysfunction in the CNS leading to demyelination and gliosis with subsequent release of inflammatory cytokines and chemokines. This cascade of events compromises the integrity of the BBB resulting in permeability to peripherally circulating T- and B-cells. Within the CNS, T-cells (CD4^+^ and CD8^+^) interact with antigen presenting cells, via major histocompatibility complex, and become activated. These activated T-cells also release inflammatory mediators such as cytokines, nitric oxide, and glutamate, which exacerbate the degenerative process leading to a subsequent immune response which further accelerates oligodendrocytosis and demyelination [adapted from (11)]. Figure was constructed using CorelDraw-version 2018 (www.coreldraw.com, Ottawa, ON, Canada) image processing software.

Two opposing hypotheses have been proposed to explain the pathoetiology of MS: “outside-in” and “inside-out” (10–12). Figure 1 summarizes these hypotheses of MS pathoetiology. In this Perspective, we first outline these hypotheses, explain their compatibility to the pathoetiology of MS, and examine how well these hypotheses are aligned with the outcomes of pre-clinical and clinical research. Finally, we show how recent advances using the cuprizone (CPZ) animal model support the inside-out hypothesis of MS pathoetiology, and how this research can be used to further investigate the earliest stages of demyelination, as well as the subsequent involvement of the adaptive immune response in aggravating the demyelinating lesions.

“Outside-in” theorists propose that an undefined dysregulation of the peripheral immune system and BBB leads to an autoimmune response against myelin components in the CNS. The central concept of and support for this hypothesis have been extensively drawn from studies using an animal model of experimental autoimmune encephalomyelitis (EAE). The EAE model was developed from a sequence of observations. The initial concept of EAE came from several observations of clinical interventions. First, 1885 rabies vaccinations to humans were shown, by 1888, to cause paralysis (13). Likewise, in 1925, repeated inoculation of humans with rabbit spinal cord homogenates was shown to cause paralysis (13). Later, these observations motivated neurologists and researchers to investigate the effect of inoculating live animals with CNS extracts [e.g., spinal cord homogenates; (13)]. In the original article “*Observations on attempts to produce acute disseminated encephalomyelitis in monkeys*,” the introduction revealed prevailing concerns about neurological disease and immunization and prophetically noted that “*The etiology of this malady is unknown in spite of the fact that considerable work has been done to disclose it*” (14). This study demonstrated that peripheral inoculation of monkeys with whole rabbit brain homogenate (injected intramuscularly) can induce a central glial reaction, loss of myelin sheaths, and “*cellular infiltration into the CNS*” (14). By 1934, a “*specific antigenicity of homologous brain runs parallel to the myelin content of the tissue*” was described and led to the speculation of post-infection encephalitis (15). In 1947, peripheral inoculation with brain homogenates in rabbit and rhesus monkey when combined with complete Freund adjuvant [paraffin oil plus heat-killed tubercle bacilli; (16)] resulted in a distribution of lesions with a “*distinct resemblance to that in disseminated encephalomyelitis and multiple sclerosis of human beings*” (17). It was also demonstrated that “*the perivascular position of the lesions, more particularly about venules and veins, is much like that in disseminated encephalomyelitis*” (17). Notably, the occurrence of relapse and remission cycles were similar to those seen in human MS (17). This model then underwent a progressive renaming from allergic encephalomyelitis (18) to EAE following the identification of the peripheral immunogenic role of exogenous human and guinea pig myelin basic protein (19, 20). The immunogenic/antigenic role of myelin basic protein may account for the over-/misinterpretation of the EAE model that has led to the widespread correlation of this model with MS rather than acute disseminated encephalomyelitis (ADEM). The correlation between EAE and the autoimmune aspects of MS has generated over 13,000 articles (https://pubmed.ncbi.nlm.nih.gov/2020) in the current form of EAE (mainly in mice) that relies on peripheral immunization with exogenous (human) antigens (e.g., myelin basic protein). Proponents of the EAE model, particularly with regard to immune cell involvement, have thus focused extensively (if not almost exclusively) on the immunological elements and argued for an (almost purely) immunological pathoetiology of MS [e.g., (21–23)]. Moreover, there is limited evidence (24) in EAE as to how autoantigens that originate in the CNS are recognized by the peripheral immune system. However, recent evidence indicates that CNS inflammation in EAE may be regulated by the meningeal lymphatic vasculature (24). Meningeal lymphatics assist in the drainage of cerebrospinal fluid-derived soluble molecules and immune cells into the lymph nodes. The ablation of meningeal lymphatics reduces the inflammatory response of brain-reactive T-cells in EAE, suggesting that drainage contributes to the activation of encephalitogenic T-cells in the lymph nodes (24). The selectivity of the immunological response to myelin elements within the CNS was questioned in studies showing that EAE also resulted in disruption of myelin structure in the peripheral nervous system and induced changes in sensory-motor functions (25–28). However, in MS, peripheral nerve involvement is limited to ~5% of patients with chronic inflammatory polyneuropathy that is responsive to corticosteroid therapy (29). In this regard, the cross-reactivity of EAE with myelin in the peripheral and central nervous systems, which resembles only a very small subset of MS patients, may well reflect immunological aspects that are shared between EAE and ADEM rather than reflecting the pathoetiology of MS *per se*.

Arguments in support of the outside-in hypothesis have focused on the presence of T-cells, activated glial cells, and focal demyelination (types I–II, Box 1) in autopsy specimens from patients with *advanced* MS (30). It thus remains uncertain whether or not the focus on the immune aspects actually addresses the *primary* causes of disease initiation (i.e., pathoetiology), as inflammatory cells are evident in both demyelinated and non-demyelinated sites, indicating that the presence of inflammatory cells alone is insufficient to induce demyelination (31). Likewise, a detailed histological analysis of MS lesions demonstrated that “*1/3* of *CNS brain lesions did not involve peripheral inflammatory cells such as T-cells*” suggesting an alternative pathoetiology of inflammation and demyelination (30). Specifically, MS lesions were characterized by extensive oligodendrocyte dystrophy and glial activation with little or no lymphocyte involvement, as seen in type III–IV lesions [Box 1; (30)]. Likewise, autopsy samples from a highly progressive MS patient (Marburg type) showed marked demyelination and glial activation in the CNS (32). In contrast, subtle perivascular infiltration of lymphocytes in the CNS and detection of few lymphocytes in the cerebrospinal fluid without any oligoclonal band were found in a 27-year-old woman (32). Additionally, autopsy samples from patients with acute MS showed nominal T- and B-cell involvement in the lesions, yet with marked oligodendrocyte loss and glial activation (33). Although MS lesions are heterogeneous (e.g., early, late, acute, or chronic), the staging or timing of MS lesion initiation and progression remains elusive (34).

Box 1 **Key summary of the heterogeneity of MS lesions in the CNS based on oligodendrocytes loss, immune cell involvement, demyelination, and BBB disruption using histological examination [adapted from (30)].**

**Patterns I and II:**

Similarities:

- Disruption of BBB which is confirmed by the presence of immunoglobulin G
- Plaques are centered by small veins and demarcated edges with peri-venous extensions
- Presence of remyelination shadow plaques (incomplete remyelinated lesions)
- Presence of T-lymphocytes and macrophages
- Less oligodendrocyte damage than in patterns III and IV
- A similar pattern of loss of myelin proteins (e.g., myelin basic protein)

Differences:

- Pattern I is found mainly in acute MS (patients who die or are subjected to biopsy within the first year after disease onset) whereas pattern II occurs mostly in chronic, relapsing-remitting MS, secondary progressive MS, and primary progressive MS
- Deposition of complement complex (part of the immune system promoting phagocytosis and inflammation) is found only in pattern II lesions

**Patterns III and IV:**

Similarities:

- Absence of remyelination shadow plaque
- Presence of T-lymphocytes and macrophages
- Large numbers of oligodendrocytes damaged
- Demyelinating lesions are around inflamed blood vessels
- Absence of immunoglobulin G and complement complex

Differences:

- Preferential loss of myelin-associated glycoprotein in pattern III
- Pattern III lesions are mostly found in patients with acute MS whereas pattern IV is found in primary progressive MS with prominent cerebellar and brain stem involvement.

These observations indicated that oligodendrocyte loss preceded the involvement of autoreactive T- or B-cells in the lesion site, i.e., *oligodendrocyte loss is an early disease event that can trigger (or otherwise be followed by) a subsequent autoimmune response*. Moreover, these observations highlighted the question as to what aspect of MS is being studied in EAE models given that the autoimmune response in humans occurs spontaneously, whereas in EAE the immune response is initiated in the periphery by the acute administration of relatively large amounts of exogenous antigens and immune activators. Additionally, there are notable differences in the cellular immune responses of MS and EAE: first, CD8^+^ T-cells are the predominant immune cells in the CNS lesions of MS patients whereas, in EAE, CD4^+^ T-cells predominate. Second, the demyelinating lesions in EAE are mainly located in the spinal cord, whereas in MS patients, lesions are mainly in the cerebral and cerebellar cortices [reviewed in (35–37)]. Third, there is a limited overlap of proteins and genes identified as being involved in EAE and MS (38, 39). Fourth, when ablation of the immune system in MS patients was followed by autologous haematopoietic stem cell transplantation, a reduction in the autoimmune response was observed but disease progression continued (40). That same study also demonstrated that over 74% of MS lesions in patients who received autologous stem cell transplantation had an active or complete demyelination with <26% of lesions showing signs of remyelination (40); furthermore, there was little T- or B-cell involvement indicating that these lesions did not originate due to an autoimmune response. However, recent work using bone marrow transplants in patients with primary progressive MS and secondary progressive MS showed reduced neurological dysfunction, better survival rates [i.e., longer lives; (41)], and a significant reduction in relapses (42), but the disease still progresses. Moreover, transplant-related mortality and exacerbation of neurological disabilities are not uncommon (41, 43, 44).

For how long can these marked differences between EAE and MS be ignored before we question the merit of accepting a correlation between an acute, peripherally-induced immune response and central demyelination (as seen in EAE) as an effective model for the *pathoetiology* of MS? It has been taken *ipso facto* as proof that a similar process underpins the initiation of EAE and MS, rather than a demonstration that EAE models a later immunological response to an earlier (and likely longer-term) oligodendrocytosis (i.e., degeneration or loss of oligodendrocytes resulting in subsequent demyelination). The almost overwhelming focus on EAE has thus allowed the “*tail to wag the mouse*” rather than the more realistic circumstance in which effect follows cause. In this scenario, MS (unlike EAE) is not a disease that is initially mediated by the T-cells trafficking from the periphery but is *caused* by another mechanism, namely “*degeneration of oligodendrocytes*” (11, 45). This must be the target if we are to understand the origins of the disease and effectively target a potential cure, a task from which we may have been diverted by focusing on EAE.

Despite nine decades (1930–2020) of intensive research, EAE has neither revealed a reliable early biomarker for MS nor aided in the development of a therapeutic that fully and effectively halts MS. Notably, EAE has most certainly advanced our understanding of the autoimmune aspects of MS, albeit later in the “clinical” stages of the disease (22, 46). Thus, several medications (e.g., glatiramer acetate, mitoxantrone, natalizumab), that are used for the symptomatic treatment of MS were developed after showing promising effects in EAE (47). Nevertheless, while these immunosuppressive therapies are not without the risk of significant side-effects, they can reduce the severity of the autoimmune attacks in some patients (e.g., decreasing the number of relapses in relapsing-remitting MS). However, they have little or no effect during the progressive phase (e.g., primary progressive and secondary progressive MS) of the disease (48, 49) suggesting alternative mechanism(s) underpinning the pathoetiology and/or disease progression of MS.

Due to the inability of EAE to address the initial, underlying pathoetiology of MS, recent studies have instead focused on the “inside-out” hypothesis of MS (50–52). This hypothesis argues that MS is initiated in the CNS as a degeneration of oligodendrocytes that subsequently triggers an innate immune response involving microglia and astrocytes, which act as antigen-presenting cells. This likely slow (i.e., perhaps over decades) degradation of oligodendrocytes results in the release of antigenic myelin proteins, such as myelin basic protein, into the circulatory and lymphatic systems, that trigger a subsequent autoimmune response in which peripheral T- and/or B-cells are activated and migrate into the CNS (10, 11). The inside-out hypothesis is consistent with autopsy findings showing extensive oligodendrocyte loss in MS patients (disease duration ~39 months) with histologically proven active lesions (30). But notably, at the early time points (acute lesions with disease duration ~20 days) of disease presentation, few inflammatory parenchymal T- and B-cells were found at the active demyelinating sites in the brain stem of MS patients (33, 53). Qualitative and quantitative magnetic resonance imaging and histopathologic analysis of the brains of chronic (disease duration ~20 years), secondary, and primary progressive MS patients showed demyelination, extensive axonal loss, and chronic gliosis rather than focal inflammation (54). In the early clinical stages of MS (e.g., 2 weeks to 8 months following clinical diagnosis), electron microscopic studies have shown that myelin degeneration or abnormal changes start at the innermost layers of the myelin sheaths, even at sites that are remote to the inflammatory CNS lesions. This suggests that the process of demyelination is not inevitably linked to inflammation and immune autoreactivity (55). Additionally, inadvertent detection of multifocal periventricular or juxta-cortical lesions in patients with non-neurological symptoms such as tumor, intracerebro ventricular bleeds, or headaches indicated the presence of lesions prior to the onset of clinical symptoms of MS (56). Likewise, samples analyzed from live MS patients also revealed less association of T- or B-cells in MS as seen in post-mortem samples (57–65). For example, changes of metabolites (e.g., a 30–40% increase of choline lipid) in normal-appearing white matter from MS patients are observed (measured by magnetic resonance *spectroscopic* imaging) before demyelinating lesions are detected using magnetic resonance imaging (65). When cerebrospinal fluid, blood, tear, or urine samples from live MS patients were analyzed few T-cell markers were detected compared to a host of other protein changes (57–64). For example, marked changes in apolipoprotein, ceruloplasmin, creatinine kinase, superoxide dismutase formed a major cluster of metabolic pathway changes in MS patients (57–64).

A genome-wide association study from 1,470 MS cases, including ~2.5 million single-nucleotide polymorphisms revealed no single genome-wide significance indicative of MS susceptibility loci influencing MS severity [International Multiple Sclerosis Genetics Consortium (66)]. However, bioinformatic (KEGG pathway) analysis showed the enrichment of genes such as Ptprd, Ywhag, or Ifna16 from different pathways including natural killer cell-mediated cytotoxicity and Wnt signaling [regulates key aspects of organogenesis, neural patterning, cell migration (67)] may contribute to the perturbation of not only immune function but also extend to oligodendrocyte degeneration [International Multiple Sclerosis Genetics Consortium; (66)]. For example, overexpression of interleukin-2 (68) or natural killer cells (69) has been shown to evoke oligodendrocyte degeneration. Moreover, dysregulation of the Wnt signaling cascade impedes oligodendrocyte differentiation and maturation resulting in impaired myelination (70, 71) by modifying energy metabolism (72). Likewise, another genome-wide association study from MS patients revealed dysregulation of genes involved in immune regulation and metabolism (73). In addition, neuronal DNA damage (measured using γH2A.X as a marker) leads to abnormal cell cycle re-entry of oligodendrocytes (using cyclin D1 as a cell cycle marker) resulting in their death in MS (74). This evidence suggests that oligodendrocyte degeneration in MS is more heterogeneous than currently perceived and both metabolic dysregulation and genetic predisposition may contribute to disease initiation and progression (75).

Radiological abnormalities (identified in T2-weighted brain magnetic resonance imaging as hyperintense foci) are detectable in clinically asymptomatic relatives (e.g., parents, siblings, or children) of MS patients, suggesting that shared susceptibilities can manifest as radiological markers (76) which may indicate structural changes in myelin and infiltration of immune cells (77, 78). While such individuals may be clinically asymptomatic, they do display tell-tail signs of changes in sensory functions, such as poorer vibration perception, suggesting that there are underlying functional changes (35, 76). Furthermore, longitudinal sampling in military personnel revealed that the serum level of neurofilament light chain (a marker of axonal degeneration) was significantly elevated up to 6 years prior to the onset of clinical MS symptoms (79). Likewise, a 30-month longitudinal study revealed axonal injury (measured by proton magnetic resonance *spectroscopy*) in the “normal-appearing” white matter where magnetic resonance imaging was unable to show any changes (80). The interplay between the axon and its supporting oligodendrocytes was evident when the loss of myelin-associated glycoprotein and apoptotic-like oligodendrocyte destruction (as seen in type III MS lesions, Box 1) were seen at the inner layers of the myelin sheath whereas the outer layers, which are more readily accessible to interact with the immune system, were unaffected (81). Overall, a substantial body of data indicates that inherent early and long-term oligodendrocyte malfunction is the primary driver of demyelination, rather than autoimmune responses.

In MS patients, it is clear that adaptive immune cells do play a key role in the longer-term, clinically-defined pathology of the disease. However, the most important question is whether these immune cells instigate the primary pathology (as in the outside-in hypothesis) or represent a normal immune response directed against a preceding oligodendrocytosis/cytodegeneration (as in the inside-out hypothesis). If the latter hypothesis is correct, then the key is to identify the underlying mechanism(s) of primary oligodendrocyte death (i.e., oligodendrocytosis). For now, this is still uncertain, but there is growing evidence arising from research using the non-immune CPZ model of a demyelination mechanism based primarily on metabolic susceptibility (50, 82, 83).

This model is generated by feeding CPZ to rodents, resulting in oligodendrocytosis and subsequent CNS demyelination and gliosis (35, 82, 84, 85). In this model, CPZ also induces atrophy of the peripheral immune organs (e.g., spleen and thymus) and thus reduced T-cell levels, thereby suppressing the functions of the adaptive immune system. Therefore, the demyelinated lesions in the CNS of the CPZ-fed animals do not involve adaptive immune cell [i.e., T-cell; (82)]. In addition, as the BBB remains intact in the CPZ model this further reduces the capability of T-cells to infiltrate the CNS (82, 86), although the lymphatic system remains functional. While some have interpreted this lack of adaptive immune involvement as a shortcoming of the CPZ model, this was largely based on the longstanding dogma developed from the use of the EAE model. In fact, what the CPZ model demonstrates in the first instance is very selective and progressive MS-like damage without the involvement of the peripheral immune system (35, 85, 87). The importance of this observation was nonetheless downplayed in the literature for quite some time. Nonetheless, the CPZ model has now been further refined using strategic modifications that either boost the immune system following cessation of CPZ-feeding (50) or protect the peripheral immune system (51) thereby resulting in progressive demyelination, local inflammation (micro/astrogliosis), and subsequent CD8^+^ T-cell infiltration into the CNS (inside-out response) when the BBB is breached.

## CPZ Model: Relevance to Modeling MS

It is commonly considered that mature oligodendrocytes are susceptible to CPZ toxicity that induces mitochondrial oxidative stress (82, 88–90). Some authors have argued that CPZ can be used as a model to explore the mechanisms involved in the later, rather than early, disease stages of MS (36, 91). However, we, and others, have reported that CPZ can be used to investigate both aspects of the disease. For example, when CPZ is fed for a short period or at a low dose (e.g., 0.1%), significant oligodendrocytosis, demyelination, and accelerated remyelination are observed (51, 82, 92–94). In addition, oligodendrocyte degeneration (95) and glial responses (96) can be observed before detectable demyelination in the CPZ model. However, prolonged CPZ-feeding (e.g., 0.2% for 12 or more weeks) leads to a progressive ablation of oligodendrocytes, massive demyelination, and axonal injury (97–99). These data suggest that CPZ can be used to investigate both early and progressive stages of MS by titrating the dose and duration of CPZ-feeding.

Although there is limited evidence of successful translation, unlike EAE, of a remyelinating drug tested in CPZ and approved for human use (100), encouragingly, a large number of therapeutics are now being tested in the CPZ model for generating drugs to promote remyelination. For example, in 2020, 25/70 published papers have investigated different medications (PubMed by July 2020). However, studies using the CPZ model to investigate drug development started just over a decade ago (101) compared to EAE where efforts began over 70 years ago (102). Some authors have argued that both EAE and CPZ are important for investigating the different aspects of MS disease (36, 91, 103). To develop therapeutics against the autoimmune response in MS, the EAE model may be used since it is immune-driven (22, 46), if we accept that the disease process is mediated by CD4 T-cells. In contrast, to promote new myelin formation (demyelination is proven as the hallmark of MS) and minimize progressive degeneration, use of CPZ would be the best choice (100). However, perhaps the more important point with CPZ is that it may better reflect the preclinical stages of the disease [or perhaps the milder form of MS (104)], and thus its potential contribution to identifying much earlier, fundamental drug targets that might fully halt the disease process (and that could thus also be used as an adjunct to the current immune-related therapeutics).

Oligodendrocyte degeneration is followed by demyelination and activation of microglia and astrocytes, resulting in gliosis. This gliosis is distributed throughout all parts of the CNS but it is most marked in the cerebrum and cerebellum compared to the brain stem and spinal cord (82, 84, 96, 105). However, a recent study demonstrated that CPZ-feeding does not induce the degeneration of mature oligodendrocytes in the spinal cord (84). With only three studies (84, 106, 107) examining the effects CPZ on the spinal cord, using varying techniques in different mouse strains it may well be premature to assert a single action. For example, 4-weeks of CPZ-feeding in C57BL/6 mice appeared to have no impact on the luxol fast blue staining and immunodetection of myelin basic protein but did reduce mitochondrial complex IV abundance in the spinal cord; gliosis was not investigated (106). In SJL mice, TUNEL-positive apoptotic cells were found in the white matter of the spinal cord with reduced NogoA (a mature oligodendrocytes marker) and myelin basic protein mRNA expression; whereas no changes was observed in C57BL/6 mice (107). However, none of the mouse strains showed demyelination and glial activation (107). In contrast, in our recent study, 5 weeks of CPZ-feeding was associated with astro- and microgliosis in both the gray and white matter of the spinal cord (84). While preliminary evidence of reduced myelin basic protein staining (unpublished data), suggested demyelination, this was not confirmed using Silver staining (84). Whether the limited demyelination in the spinal cord of the CPZ model is due to technical issues [e.g., saturation of Silver staining in the high-density tracts of the spinal cord (*vis-a-vis* low-density tracts where demyelination was readily shown) (84)] or the use of different staining methods (e.g., Silver, myelin basic protein) remains unclear.

The apparent limited demyelination in the spinal cord in CPZ, unlike that seen in EAE, is less consistent with MS in which spinal cord lesions are seen, but less so than in the brain (108–111). However, the detection of spinal cord demyelination in humans is technically demanding (due to the thin cord, cerebrospinal fluid, bone, fat) with conventional imaging techniques and may go undiagnosed during asymptomatic stages (109, 110, 112, 113). Moreover, differential pathological outcomes are found in different segments of the spinal cord of MS patients; for example, lesions are more common in the cervical (~60%) than the thoracic or lumbar spinal cord (114, 115). In addition to brain and cerebellum lesions, we have shown demyelination and gliosis in the brain stem of CPZ-fed mice (84) which is also seen in newly forming demyelinating lesions in MS patients (33, 116).

In addition to demyelination and gliosis, we (84) and others (117) have shown comparable functional deficits as seen in human MS [reviewed in (35)]. The apparent absence of correlation between behavioral deficits and histological changes may well be attributed to an undue focus on the corpus callosum (35); our most recent study showed that early motor deficits are associated with changes in the spinal cord, brain stem, and cerebella and cortical pathways associated with sensory-motor function (84). A perhaps more controversial interpretation of the results with CPZ is that the lack of more profound physical manifestations as seen in MS indicates that the main clinical symptoms of MS are not primarily related to demyelination but rather to molecular/cellular alterations that have yet to be effectively characterized. Alternatively, it maybe that rodents are of limited utility in modeling the human disease. As always, correlations (notably with findings in animal models) are just that, and interpretations, as to “likely” causation, vary.

These observations indicate that there are regional differences in the CNS in terms of oligodendrocytosis and highlight the fact that oligodendrocytosis and inflammatory responses can occur independently of each other. However, the underlying susceptibility to the differential response of CPZ on glial cells is neither clearly understood nor been systematically investigated. The heterogeneity of the glial response may depend upon the differential expression of type III neuregulin-1 (118) and Fyn (119). For example, loss of the non-receptor tyrosine kinase Fyn (a signaling molecule of the Src kinase family) causes hypo-myelination (120) in the brain rather than the spinal cord (119). Likewise, mice haplo-insufficient for type III neuregulin-1 (a growth factor that promotes oligodendrocyte and Schwann cell development) showed reduced myelination in the corpus callosum (118). In contrast, no effect was observed in the optic nerve and spinal cord, further indicating regional differences in the regulation of OLG function and their susceptibility to injury (118). Whether the expression of Fyn or neuregulin-1 contributes to the regional heterogeneity of oligodendrocytosis in CPZ-fed animals remains untested.

Another possibility for these effects could be that different regions of the CNS have different subtypes of oligodendrocytes (types I–IV) based on biochemical profile and axon myelination (121). Most recently, RNAscope analysis showed 12 different subtypes of mature oligodendrocytes that were not only differentially distributed in the brain and spinal cord but also responded differently to injury (122). Likewise, single-cell RNA sequencing of oligodendrocyte lineage cells from 10 CNS regions (e.g., hippocampus, hypothalamus) revealed 13 distinct populations (123), suggesting the region-specific expression of oligodendrocyte lineage cells in the CNS. In addition, oligodendrocyte lineage cells from EAE spinal cord show overexpression of genes involved in antigen processing and presentation via major histocompatibility complex classes I and II (124). In contrast, oligodendrocyte progenitor cells are capable of phagocytosis and activate memory and effector CD4-positive T-cells (124)—suggesting an oligodendrocyte mediated immune response in EAE. Likewise, a similar result was found when demyelinated areas (e.g., normal-appearing white matter) were investigated using single-cell RNA sequencing from MS patients (125). This analysis showed a differential expression of RNA markers in MS patients which was either unique or enriched (125). For example, platelet-derived growth factor receptor A (pdgfra) is uniquely expressed in oligodendrocyte progenitor cells, whereas apolipoprotein E (apoE) is expressed in immune oligodendroglia (125). Whether these aforementioned factors contribute to the regional distribution of glial responses in the CPZ model requires future investigation.

Despite the marked CNS oligodendrocytosis, demyelination, and gliosis in CPZ-fed mice, no involvement of adaptive immune cells in the CNS lesions was found (82, 86). However, T-cell infiltration into the CNS was not evident even when the BBB was disrupted by injection of ethidium bromide, lysolecithin (86), or pertussis toxin (82). This indicated the presence of an alternative mechanism(s) in the adaptive immune cell response in the CPZ model, independent of BBB integrity or the lymphatic system. Recent studies have shown CPZ-induced suppression of the adaptive immune system (82, 126–129). A time-dependent reduction in the number of CD4^+^ T-cells (~50%) and an inability to detect upregulation of T-cells (using CD44 and CD69 as markers) were observed in the corpus callosum following CPZ-feeding (126). Likewise, longer observations (10 months after cessation of CPZ-feeding) did not reveal any T-cell infiltration into the CNS (52). However, it was hypothesized that CPZ might have a direct immunosuppressive effect on the adaptive immune cells (52). Other studies demonstrated that the involvement of adaptive immune cells in EAE and Theiler's murine encephalomyelitis was reduced following CPZ-feeding, resulting in a delay in the development of disease characteristics (130–132). Our recent findings have revealed that the size of the spleen, as well as its T-cell (CD4^+^ and CD8^+^) levels, were reduced in CPZ-fed animals, following both short and prolonged feeding (82), confirming previous observations (127–129). Moreover, using a top-down proteomic analytical approach, a decreased abundance of specific proteoforms (e.g., of leukocyte elastase inhibitor A, calcium/calmodulin-dependent protein kinase type II subunit alpha, and disulphide isomerase) known to be involved in T-cell function were identified (82, 133). Furthermore, a reduction of the abundance of complement protein (part of the immune system) was found in the peripheral blood mononuclear cells of CPZ-fed animals (133). These findings indicated that CPZ-induced peripheral immune system suppression would have to be overcome in order to fully address the inside-out hypothesis of MS.

The molecular basis by which CPZ-ingestion causes adaptive immune system suppression is unclear, but CPZ chelates copper, leading to dyshomeostasis of other essential ions such as iron, zinc, sodium, and manganese in organs such as the brain and liver (134–138). The reduction in T-cells following CPZ-feeding is not surprising since copper is required for the synthesis of interleukin-2, and decreased levels of interleukin-2 interfere with the growth and maturation of T-cells (139, 140). Beyond the suppressive effect of CPZ (e.g., reduction of T-cell number), studies have revealed that T-cell functionality relies on mitochondrial activity (141). In CPZ-fed mice, mitochondrial division is inhibited resulting in the formation of extremely enlarged “mega-mitochondria” in oligodendrocytes, hepatocytes, and thymocytes (128, 142–145). Mega-mitochondria formation is an abnormal process that can result in excessive amounts of reactive oxygen species which reduce adenosine triphosphate supplies leading to cellular energy failure (146). Moreover, CPZ interferes with the fission and fusion dynamics of mitochondria due to the reduction of abundance of dynamin 1 protein (82) leading to the progressive swelling of mitochondria (88), reduction of mitochondrial transmembrane potential (90), and reduction of nicotinamide adenine dinucleotide metabolism (147). In addition, gene expression analysis revealed marked mitochondrial gene changes in CPZ-fed mice (148). Likewise, a selective loss of mitochondrial complex IV was found in cerebellar Purkinje neurons following CPZ-feeding for 5 weeks (149). Moreover, a marked decrease in the activities of mitochondrial complexes I-III in the brain of CPZ-fed mice was also found (150). Furthermore, the addition and deletion of mitochondrial DNA have been shown in CPZ-fed rats (151). These changes in mitochondrial function are also supported by human MS studies; for example, microarray analysis of post-mortem motor cortices from MS patients revealed the downregulation of nuclear-encoded mitochondrial genes and decreased activity of mitochondrial respiratory chain complexes I and III (152). This is also further supported by the elevated level of mitochondrial stress markers in the serum of MS patients (153). Moreover, a case report revealed that mutation in the DNA polymerase gamma gene is responsible for the changes in mitochondrial function and has been implicated in MS-like illness including ophthalmoplegia, ataxia, and cognitive impairment (154) – suggesting that MS may well originate as a disease of mitochondrial dysfunction (155, 156).

Furthermore, proteomic and bioinformatic analyses revealed marked changes in metabolic pathways associated with mitochondrial functions (82). Thus, dysregulation of mitochondrial processes may underlie the compromise of the peripheral immune system in the CPZ model (157). In addition, since oligodendrocytes depend upon mitochondria for energy (158), the compromised mitochondria following CPZ-feeding supply less energy to oligodendrocytes, thus triggering oligodendrocytosis (i.e., the suggested initial trigger of MS).

Can CPZ-induced immune system suppression be prevented? We have addressed this question recently (51, 82). CPZ induces atrophy of the peripheral immune organs such as the spleen, making it impossible to see whether or not T-cells can invade the CNS when the BBB is breached (82). To circumvent this problem, we used juvenile mice [to avoid age-related thymic involution (159, 160)] and fed them 0.1% CPZ for 2 weeks to overcome the normal CPZ-mediated adaptive immune system atrophy (51). Juvenile animals showed less splenic and thymic atrophy when fed with 0.1 or 0.2% CPZ (51) compared with young adult mice (82, 127–129). However, no CNS T-cell infiltration was seen after the BBB was breached using pertussis toxin (51). In a parallel study, we castrated the juvenile mice to completely prevent splenic and thymic atrophy (51), since it is known that castration overcomes androgen-dependent (age-related) thymic involution and maximizes adaptive immune cell maturation and function (161, 162). When we combined castration, 0.1% CPZ-feeding and pertussis toxin injection to juvenile mice for 2 weeks, CD8 T-cell infiltration into the CNS, in addition to demyelination and gliosis, was observed (51). The result was confirmed by western blotting, immunohistochemistry, and flow cytometry (51). This work concluded that “*CD8*^+^ *T-cell recruitment into the CNS of CPZ-fed mice, albeit castrated male mice, provides a potential new variant of the CPZ model with which to explore the early events involved in CNS demyelinating diseases like MS*” when the BBB is compromised (51). In contrast, gonadally intact female mice showed the routinely observed CPZ-induced thymic and splenic atrophy, and no T-cell infiltration into the CNS (51). It is noteworthy, however, that MS is more prevalent (2–3-fold) in females than males (163) suggesting that the lack of T-cell involvement in the CPZ-fed female mice (51) may indicate that different mechanism(s) are involved in the pathoetiology of MS in males and females. Whether female hormones (e.g., estrogen and progesterone) play a role in CPZ outcome or T-cell infiltration into the CNS remains untested. However, the preferential presence of CD8^+^ T-cells in this model more closely resembles human MS pathology since CD8^+^ T-cells outnumber CD4^+^ T-cells by 3-10-fold in MS patients (164, 165). The work of Almuslehi et al. (51) is supported by another recent observation in which the peripheral immune systems of CPZ-fed mice were boosted by peripheral injection of complete Freund's adjuvant, and the BBB was breached (50). In this model, CNS infiltration of CD3^+^ T-cells (a pan T-cell marker) was evident, and was followed by a secondary demyelination and inflammatory process. This variant of the CPZ model has been termed “cuprizone autoimmune encephalitis.” The origin of the encephalomyelitis was attributed to citrullination (50), a post-translational modification in which the amino acid arginine is converted to citrulline leading to conformational changes of the affected proteins (166). Citrullination of myelin basic protein is linked with lymphocyte infiltration and demyelination in the spinal cord in EAE (167) and MS lesions (168). Western blot analysis showed a shift in the molecular weight in the blot of peptidyl arginine deiminases which are similar in molecular weight to myelin basic protein (50). Altogether, the data indicate that metabolic dysregulation in the CNS can lead to a subsequent peripheral immune response in CPZ-fed mice.

What happens when adoptive myelin-reactive T-cells are transferred to the CPZ-fed animal? This question was addressed recently (169–171). T-cells were transferred from EAE mice into CPZ-fed mice via intraperitoneal injection and CD4^+^ T-cells infiltrated the corpus callosum; delayed remyelination was observed, suggesting that T-cells promote continuous demyelination and slowed remyelination in CPZ-fed mice (170). This model was further developed by Kirby et al. (171) who demonstrated that the adoptive transfer of myelin-reactive T-effector cells influenced the properties and differentiation of oligodendrocyte precursor cells (171). This work also showed that oligodendrocyte precursor cell differentiation is reduced by both effector T-cells and interferon-γ overexpression by astrocytes (171). Moreover, oligodendrocyte precursor cells exposed to interferon-γ cross-present antigens to cytotoxic CD8 T-cells, leading to oligodendrocyte precursor cell degeneration (171). Similarly, peripheral immunization of myelin oligodendrocyte glycoprotein 35–55 peptide into CPZ-fed mice induced myelin autoreactive T-cell infiltration into the CNS (169). All these studies indicate the importance of brain-intrinsic degenerative cascades for immune cell recruitment and degeneration of oligodendrocytes. However, histological investigation mainly concentrated on the corpus callosum (170, 171) and no reports of either behavioral deficits or proteomic changes associated with this model were found in the literature, clearly indicating that further studies are required. Moreover, these studies (169–171) relied on a preactivated anti-myelin (e.g., myelin oligodendrocyte glycoprotein) T-cell mediated immune response (arguably another variant of EAE; i.e., outside-in) rather than endogenous myelin (50–52).

New CPZ model variants (50, 51) reflect both primary oligodendrocytosis followed by the production of endogenous antigens (e.g., myelin debris) and a subsequent adaptive immune response. Whether or not T-cells were functionally active or if these treatments resulted in behavioral deficits were not tested (50, 51) and should be part of the next studies using these models. However, the microenvironment that facilitates T-cell infiltration was also not assessed in these recent studies (50, 51, 82). For example, the role of pro-inflammatory cytokines (e.g., interleukins-1 and−6, tumor necrosis factor-α, and interferon-γ) from microglia and astrocytes in the process of T-cell infiltration should be investigated (172). This would test which cytokine(s) are responsible for the peripheral T-cell activation and migration into the CNS following castration and the breach of the BBB in CPZ-fed mice. Moreover, Sen et al. recently found a number of proteoforms (e.g., of calreticulin and dynamin) that appeared to have arisen due to selective post-translational modifications following CPZ-feeding (82); however, the antigenicity of these proteoforms was not tested (82) as for the peptidyl arginine deiminases (50). While this study (82) used whole-brain samples to identify proteome changes, detailed proteomic analysis of tissue from defined regions of the CNS including the cerebellum, brain stem, and spinal cord may explain the temporal effects of CPZ.

The presence of an “oligodendrocytosis triggering immune response” in the CPZ model is also supported by studies from the diphtheria toxin model (52). In this model, targeted oligodendrocyte degeneration is achieved either via external administration (173, 174) or genetic manipulation (52, 175, 176) of diphtheria toxin in the rodent. However, oligodendrocytosis triggering immune responses depend upon the duration and nature of degeneration. For example, when animals are treated for a short period such as 4 (174), 6 (176), or 5–20 weeks (173), motor behavioral deficits, oligodendrocyte degeneration, glial activation, and axonal injury are observed, but no adaptive immune response occurred. On the contrary, longer incubation leads to immune-mediated oligodendrocyte degeneration (52). In this work (52), ~30 weeks after recovering from oligodendrocyte loss and demyelination, a secondary disease progression was observed which included motor behavioral deficits, weight loss, demyelination, and axonal injury. Importantly, this late-onset disease was also associated with increased numbers of T-lymphocytes in the CNS and myelin oligodendrocyte glycoprotein-specific T-cells in lymphoid organs (52). These data suggest that progressive degeneration of oligodendrocytes triggers an adaptive autoimmune response against myelin (52) and this is arguably more consistent with the apparent slow progression to MS (i.e., the disease, like other neurodegenerative diseases, may initiate years before the condition is clinically diagnosed).

While the diphtheria toxin model shows the sensory-motor behavioral deficits (52, 173, 175–177), these deficits do not extend to cognitive, affective (anxiety) or visual modalities like those seen in MS and the CPZ animal model (35). Moreover, studies revealed diphtheria toxin-mediated oligodendrocyte death and gliosis in the absence of a peripheral autoimmune response (173, 176, 177). Whether this effect is due to the effect of diphtheria toxin on peripheral immune organs, such as the spleen or thymus remains untested. Furthermore, research from our lab (82, 133) and others (89, 129, 178, 179) revealed similarities and differences of proteomic changes in MS and CPZ that remain unquantified in the diphtheria toxin model. In addition, the novel approaches adopted in the past 5–10 years of combining CPZ and EAE (169–171) and diphtheria toxin-induced oligodendrocytosis (52, 173–177) compared to the over 70 years of research on EAE (14) and ~60 years on CPZ (180) have yet to reveal their role in promoting a better understanding of the pathoetiology of MS.

Taken together, evidence from animal models (50–52) thus support the proposed initial inside-out pathoetiology of MS (10, 11). While with diphtheria toxin it takes ~1 year to see adaptive immune cell recruitment (52), in the CPZ model, this adaptive immune cell recruitment to the site of demyelination and subsequent immune-mediated demyelination is seen as early as 2 weeks after the start of CPZ-feeding (50, 51). Ideally, while it is quite unequivocal to state that there is no perfect animal model (perhaps for any human diseases) that mimics the complete complexity of MS, the legitimate use of an animal model depends upon the research question to be addressed and a considered presentation of the findings that acknowledges the limitations of the model. Having said that, significant progress in our understanding of MS has been made using animal models and it is our consistent hope that healthy debate, as presented in this Perspective, leads to better and more revealing experiments.

## Conclusions

This Perspective describes why the EAE model is widely used to study the late (autoimmune) aspects of MS. Since, by design, EAE supports the outside-in hypothesis, it is thus not an effective model to study MS pathoetiology and should not be the model of choice when experiments are designed to identify the initiating trigger(s) of MS. We thus argue that MS primarily originates from a slow, progressive oligodendrocyte degeneration caused by metabolic dysfunction that leads to subsequent reactive gliosis in the absence of adaptive immune cell response. The CPZ model supports the inside-out hypothesis of MS pathoetiology, and can be modified to also study CNS infiltration of peripheral immune cells. The CPZ model thus has the advantage of enabling the study of oligodendrocytosis in the absence (immuno-suppression) and presence (immuno-protection) of the peripheral immune system via titration of the dose and time. However, the full extent to which adaptations of the CPZ model mimic human MS pathology is still unclear but appropriate studies into the potential underlying/initiating pathological alterations are now possible. Initially, this will require further optimization of the new models (e.g., confirming the functionality of T-cells and testing for behavioral changes) and thus potential validation as appropriate systems for identifying novel early biomarkers and therapeutic targets to most effectively address and perhaps even cure MS.

## Data Availability Statement

The original contributions presented in the study are included in the article/supplementary material, further inquiries can be directed to the corresponding author.

## Author Contributions

MS and DM conceived the idea and drafted the manuscript. MA, PS, and JC reviewed the manuscript. JC initiated the MS research project at WSU upon which this paper builds. All authors approved the final version.

## Conflict of Interest

The authors declare that the research was conducted in the absence of any commercial or financial relationships that could be construed as a potential conflict of interest.
